# Integrins β1 and β3 are biomarkers of uterine condition for embryo transfer

**DOI:** 10.1186/s12967-016-1052-0

**Published:** 2016-10-26

**Authors:** Guowu Chen, Aijie Xin, Yulin Liu, Changgen Shi, Junling Chen, Xiaofeng Tang, Ying Chen, Min Yu, Xiandong Peng, Lu Li, Xiaoxi Sun

**Affiliations:** 1 Obstetrics and Gynecology Hospital of Fudan University, Shanghai, 200011 China; 2 Shanghai Ji Ai Genetics and IVF Institute, Obstetrics and Gynecology Hospital, Institute of Reproduction and Development, Fudan University, Shanghai, 200011 China; 3 Shanghai Key Laboratory of Female Reproductive Endocrine-Related Diseases, Obstetrics and Gynecology Hospital, Fudan University, Shanghai, 200011 China; 4 China National Population and Family Planning Key Laboratory of Contraceptive Drugs and Devices, SIPPR, Shanghai, 200032 China

**Keywords:** Biomarkers, Uterine receptivity, Integrin β3, Integrin β1, Concatenated progesterone effect

## Abstract

**Background:**

Clinical ovulation induction induces blood estrogen (E_2_) in excess of physiological levels, which can hinder uterine receptivity. In contrast, progesterone produces the opposite clinical effect, suggesting that it might be capable of recovering the lost receptivity resulting from exposure to high estrogen levels. Integrins are the most widely used biological markers for monitoring uterine conditions. We studied progesterone-induced changes in integrin β expression patterns as biomarkers for changes in uterine receptivity in response to increased estrogen levels.

**Methods:**

Endometrial biopsy samples from patients were screened for their estrogen (E_2_) and progesterone (P4) content and expressing levels of integrin β1 and β3. Uterine receptivity was evaluated using human endometrial adenocarcinoma cells in an embryo attachment model. The respective and concatenated effects of embryo attachment and changes in the integrin β1 and β3 expression patterns on the adenocarcinoma cell plasma membranes in response to 100 nM concentrations of E_2_ and P4 were evaluated.

**Results:**

Increased blood E_2_ concentrations were associated with significantly decreased the levels of integrin β3 expression in uterine biopsy samples. In vitro experiments revealed that a 100 nM E_2_ concentration inhibited the distribution of integrin β3 on the plasma membranes of human endometrial adenocarcinoma cells used in the embryo attachment model, and resulted in decreased rates of embryo attachment. In contrast, P4 enhanced the expression of integrin β1 and promoted its distribution on the plasma membranes. Furthermore, P4 recovered the embryo attachment efficiency that was lost by exposure to 100 nM E_2_.

**Conclusions:**

Blood E2 and P4 levels and integrin β3 and β1 expression levels in uterine biopsy samples should be considered as biomarkers for evaluating uterine receptivity and determining the optimal time for embryo transfer.

*Trial registration* Trial number: ChiCTR-TRC-13003777; Name of registry: Chinese Clinical Trial Registry; Date of registration: 4 September 2013; Date of enrollment of the first study participant: 15 October 2013

**Electronic supplementary material:**

The online version of this article (doi:10.1186/s12967-016-1052-0) contains supplementary material, which is available to authorized users.

## Background

Assisted reproduction techniques such as ovulation induction, in vitro fertilization (IVF), and embryo transfer (ET) have enabled patients to overcome a variety of human infertility disorders. However, the implantation rates for most IVF or ET programs remain low, even when apparently healthy embryos are used [1]. Differentiation of the uterus to a receptive state, as well as the association between blastocysts and the uterine luminal epithelium play determinant roles in the embryo implantation process [2–4]. In humans, the uterus becomes “receptive” at an appropriate stage of the menstrual cycle, enabling the blastocysts to attach. This so-called “receptive window” is initially dependent on the balance between estrogen and progesterone levels. Studies in animal models have shown that estrogen is essential for preparation of the progesterone (P4)-primed uterus to become receptive [2, 5]; however, the exact mechanism by which estrogen produces its effects when interacting with P4 is poorly understood. Due to the potential clinical importance of the balance between estrogen and P4, it is reasonable to perform a comprehensive study exploring how the estrogen-P4 interaction assists in establishing uterine receptivity.

Implantation is viewed as a “receptor-mediated” phenomenon [6]; and from that perspective, it is not surprising that extracellular matrix (ECM) ligands play fundamental roles in cell–cell interactions that occur during implantation [7]. ECM and its receptors modulate numerous key physiological activities in cells, including those related to embryogenesis and fetal development [8]. Integrin proteins comprise one major class of ECM receptors, and participate in cell–cell and cell-substratum interactions. Members of the integrin family are transmembrane glycoproteins that are present on the plasma membrane and are formed by non-covalent associations between α and β subunits. Each subunit consists of an extracellular domain, an intracellular domain, and a transmembrane region. The specific participation of integrins in implantation has early been demonstrated: based on the previous studies, members of integrins play key roles in the signaling [9], maintenance of epithelial polarity [10], and developmental progression of placental cytotrophoblast to an invasive phenotype [11]. While human uterine epithelium and glandular epithelial cells express multiple integrins [8], only the secreted integrins α1β1, α4β1 and αvβ3 display periodicity. Integrin β1 is constitutively expressed during the menstrual cycle and mouse blastocytes lack of integrin β1 subunit fail to implant, which is due to the inability to adhere or to invade the subepithelial stroma [12]. Integrin β3 is expressed on a weekly basis by epithelial cells in regions of proliferative endometrium [13]. Up-regulation of integrin β3 by the blastocyst has been proved in co-cultured human endometrial epithelial cells, which might be mediated by the embryonic IL-1 system [14]. Indeed, integrins are some of the best characterized biomarkers of uterine receptivity, and the roles they play in implantation have been widely reviewed [15–17].

Based on the above information, it was reasonable for us to comprehensively study the mechanism through which integrins β1 and β3 took their action in the formation of uterine receptivity which is driven by balance transition between estrogen and P4 balance. For such purpose, a series of in vivo and in vitro assays were performed in the current study. And with findings outlined in the current study, we expected to confirm the potential of integrins β1 and β3 as biomarkers for monitoring the estrogen-P4 balance during the establishment of uterine receptivity and reveal the interaction between estrogen and P4 during the process.

## Methods

### Chemicals, cell cultures, and animals

17-β estradiol (E_2_, dissolved in ethyl alcohol), progesterone (P4, dissolved in DMSO), and 2 % gelatin solution were purchased from Sigma-Aldrich (St. Louis, MO, USA). Anti-integrin β1 (CAT. ab52971) and anti-integrin β3 (CAT. ab75872) antibodies were purchased from Abcam (Cambridge, UK). FITC labeled anti-integrin β3 antibody (CAT. 555753) was from BD Bioscience (San Jose, CA, USA). Anti-integrin β1-Alexa Fluor^®^ 488 Antibody (CAT. FCMAB375A4) was purchased from Millipore (Bedford, MA, USA). The western blotting detection reagents were from Amersham (Arlington Heights, IL, USA). All other chemicals were purchased from Sigma-Aldrich. Human endometrial adenocarcinoma cells (Ishikawa) were maintained in DMED/F12 nutrient mixture (Life Technologies, Gaithersburg, MD, USA) containing 10 % fetal bovine serum (FBS), 1 mM sodium pyruvate, 2 mM l-glutamine, 100 μg/mL streptomycin, and 100 IU/mL penicillin in an atmosphere of 95 % air and 5 % CO_2_ for 48 h prior to hormone treatment and embryo attachment. Female C57BL/6 × DBA/2 mice (aged 8–10 weeks) were purchased from Shanghai SIPPR BK Laboratory Animal Ltd., and housed in an environmentally controlled facility, with food and water available *ad librum*.

### Patients and clinical interference

When patients underwent in vitro fertilization from 2013 to 2014 at the Shanghai Jiai Genetic and IVF Institute, forty-four patients were enrolled for this study. None of them had received any hormone therapy or an intrauterine device (IUD) within 3 months prior to their biopsy procedure. Patients who had polycystic ovaries (PCO), endometriosis, endometrial polyp or any other unconformable complaints or signs were excluded. The forty-four patients were randomly assigned to three groups for further analysis. Subjects in the control group (n = 13) began using the Luteinizing Hormone (LH)-surge test paper detection method starting on day 9 of menstruation, with the day of ovulation defined as D0. These patients underwent an endometrial biopsy on D5. The remaining 31 patients received 0.05 mg Gonadotropin-Releasing Hormone (GnRHa) for 14 days, starting on day 21 of menstruation. Next, follicle-stimulating hormone (FSH) was injected to stimulate the secretion of follicles until a subsequent injection of human chorionic gonadotropin (HCG) (D0). An endometrial biopsy was collected on D5. The 31 patients were then randomly assigned to two groups on D0. Based on previous studies, an E_2_ concentration of 10 nM was deemed optimal for inducing phosphorylation of ERK1/2 and Akt, and that E_2_ level was selected as the grouping criterion in the present study [18]. Patients with an E_2_ level ≤10 nM were assigned to a normal effect group (NE group, n = 13), while patients with an E_2_ level >10 nM were assigned to an increased [E_2_] group (OP group, n = 18). Endometrial cells were collected from all patients in both groups.

### Real time quantitative PCR

For qPCR detection, the total RNA from different samples was extracted using TRIzol reagent according to the manufacturer’s instructions. GAPDH served as a reference gene. The cDNA templates for integrins β1 and β3 were created by reverse transcription of RNA using a RT-PCR kit (Fermentas; Waltham, MA, USA). The 20 μL reaction mixture consisted of 10 μL of SYS BR Primix Ex Taq 2 solution, 0.5 μL of each primer (*integrin β1*, forward primer: ATGTGTCAGACCTGCCTTGG, reverse primer: GGGACACAGGATCAGGTTGG; *integrin β3*, forward primer: GGCAAGTGTGAATGTGGCAG, reverse primer: GACTCAATCTCGTCACGGCA; *GAPDH* forward primer: 5′-TATGATGATATCAAGAGGGTAGT, reverse primer, 5′-TGTATCCAAACTCATTGTCATAC-3′), 1 μL of the cDNA template, and 8 μL of RNase-free H_2_O. The amplification conditions were as follows: a denaturation step performed at 95 °C for 10 min, followed by 40 cycles at 95 °C for 15 s, 60 °C for 1 min, and 72 °C for 30 s. The relative integrin β1 and β3 expression levels were calculated using Data Assist Software version 3.0 (Applied Biosystems/Life Technologies; Carlsbad, CA, USA) and the 2^−∆∆ct^ method.

### Treatment of Ishikawa cells with E_2_ and P4

Ishikawa cells were seeded into six-well plates (2 × 10^5^ cells/well) and incubated with culture medium for 24 h. Following culture, the medium was aspirated and replaced with phenol-red free medium supplemented with 2.5 % CS-FBS. Forty-eight hours later, the cells were treated with E_2_ (0, 0.1, 1, 10 and 100 nM), P4 (0, 0.1, 1, 10 and 100 nM) or E_2_ plus P4 (0–0, 100–0, 100–10 and 100–100 nM, respectively) for 16 or 48 h. Each treatment group consisted of three replicates.

### Western blotting assay

Ishikawa cells which had received the different treatments were scraped from the six-well plates and lysed with RIPA lysing buffer (Beyotime; Nantong, China) containing 1 mM PMSF and a protease inhibitor cocktail (Beyotime). The lysed cells from each treatment group were centrifuged at 16,000*g* for 20 min, and the supernatant fractions were collected. Next, a 20 μg sample of supernatant protein was separated by 8 % SDS-PAGE, and then semi-dry blotted onto PVDF (polyvinylidene fluoride) membranes (Millipore, Bedford, MA, USA). After being blocked for 2 h with TBST containing 5 % non-fat dry milk, the membranes were incubated overnight at 4 °C with a primary rabbit monoclonal antibody to integrin β1 (1:5000) or integrin β3 (1:5000); after which, they were incubated with the HRP-labeled secondary antibody (1:10,000; Cwbiotech, Beijing, China) for 1 h at room temperature. HRP-labeled β-actin (1:10,000; Sigma-Aldrich) was used as an internal control protein. The protein blots were developed using Beyo ECL Plus reagent (ThermoFisher Scientific; Waltham, MA, USA), and the results were recorded with a gel imaging system. The relative expression levels of integrins β1 and β3 in the different samples were calculated using a Gel-Pro-Analyzer (Media Cybernetics; Rockville, MD, USA).

### Flow cytometry

The distribution and relative amounts of integrins located on the surface of Ishikawa cells were analyzed by flow cytometry. After 16 h of hormone treatment, the starved Ishikawa cells were scrapped off of their culture dishes, washed with PBS, and adjusted to a concentration of 1 × 10^6^ cells/mL. Next, an aliquot containing 1 × 10^6^ cells was labeled with FITC-labeled anti-integrin β3 antibody (1:100) or Anti-Integrin β1-Alexa Fluor^®^ 488 Antibody (1:50) in the dark for 1 h. After labeling, the cells were washed and resuspended in 200 μL of PBS; after which, the distribution and amount of integrins on their surfaces were analyzed using a BD FACSCanto II system (BD Biosciences; Franklin Lakes, NJ, USA).

### Ovulation induction and embryo collection

Female C57BL/6 × DBA/2 mice were superovulated by intraperitoneal injection with 10 IU of pregnant mare serum gonadotrophin (PMSG). At 46–48 h post-PMSG injection, 10 IU of human chorionic gonadotrophin (HCG) was injected; after which, each female mouse was housed with male mice overnight. The presence of a vaginal plug the following morning was defined as an indicator of successful mating. After mating, two-cell embryos (1.5 dpc) were collected from the oviduct of each mouse under a microscope. The flushed embryos were washed with M2 medium (Sigma-Aldrich) containing 4 mg/mL BSA, and then transferred into a 60 μL drop of KSOM medium (Millipore) for further washing. After washing in KSOM, the embryos were cultured in a 30 μL drop of KSOM covered with mineral oil, and incubated at 37 °C in an atmosphere of 5 % CO_2_ until reaching the blastocyst stage. Only expanded blastocysts (3.5 dpc) with normal morphology were used in further experiments.

### Analysis of embryo attachment

E_2_ (0, 10 and 100 nM) or E_2_ and P4 at different concentrations (100–1, 100–10 and 100–100 nM) was added to starved Ishikawa cells being cultured in gelatin-coated 12-well plates. Next, based on the total number of blastocytes recovered, five to 15 randomly selected blastocysts (3.5 dpc) were transferred into a hormone-treated well and incubated for 48 h; after which, the ability of the embryo to attach to the well was determined by microscopic examination using a previously published method [19]. Three samples of each treatment group were examined for attachment.

### Statistical analysis

Statistical analyses were performed using GraphPad Prism 5.0 software (GraphPad Software Inc; La Jolla, CA, USA) and IBM SPSS Statistics for Windows, Version 19.0. Armonk, NY: IBM Corp. Data are expressed as the mean ± SE or mean ± SD. Multiple means were compared using the two-tailed and paired T tests. *P* < 0.05 were considered statistically significant.

## Results

### Patient demographic information and the effect of HCG treatment on E_2_ and P4 production

The three groups of patients were not significantly different in their conformable complaints, mean age, mean Body Mass Index (BMI) value, clinical signs or basal levels of E_2_, P4, LH, and FSH (Table 1; Additional file 1: Table S1). The levels of E_2_ and P4 were dramatically up-regulated following administration of HCG (Table 2). As described in “Methods” section, the patients were divided into two groups based on their level of E_2_. Patients in the normal- and increased-E2 groups showed no significant difference in their P4 levels.

**Table 1:** Characteristics of the women in natural and stimulated cycles

Group		COH cycle		
		Control group (Ctr; n = 13)	Normal effect group (NE; n = 13)	Over physiological group (OP; n = 18)
Age (year)		30.77 ± 1.29	28.54 ± 0.81	28.61 ± 0.69
BMI (kg/m^2^)		22.56 ± 0.87	21.19 ± 0.84	20.65 ± 0.76
Basal level				
E_2_ (pM)		126.19 ± 12.52	130.43 ± 13.48	145.58 ± 16.46
P4 (pM)		0.67 ± 0.10	0.75 ± 0.11	0.49 ± 0.10
LH (mIU/ml)		4.85 ± 0.54	3.55 ± 0.49	4.35 ± 0.32
FSH (mIU/ml)		8.19 ± 0.63	6.78 ± 0.48	7.62 ± 0.42
T (ng/dl)		38.00 ± 3.43	37.38 ± 2.15	36.61 ± 3.01
PRL (ng/ml)		17.86 ± 3.14	20.21 ± 2.41	16.17 ± 1.90
GN			22.12 ± 1.14	22.48 ± 1.48

Values are mean ± SD

*BMI* body mass index; *GN* gonadotropin; *COH* controlled ovarian hyperstimulation

**Table 2:** The hormon level of the patients at day 5 after HCG injection

	HCG administration	
	Normal effect group (NE; n = 13)	Over physiological group (OP; n = 18)
E_2_ (pM)	6545.02 ± 527.64	20,722.12 ± 1426.15**
P4 (pM)	1.15 ± 0.18	1.55 ± 0.27
LH (mIU/ml)	4.75 ± 0.98	2.74 ± 0.63

Values are mean ± SD, Group NE vs Group OP

** P < 0.01

### Inhibition of integrin β1 and β3 expression was associated with increased E_2_ level

**Fig. 1:**
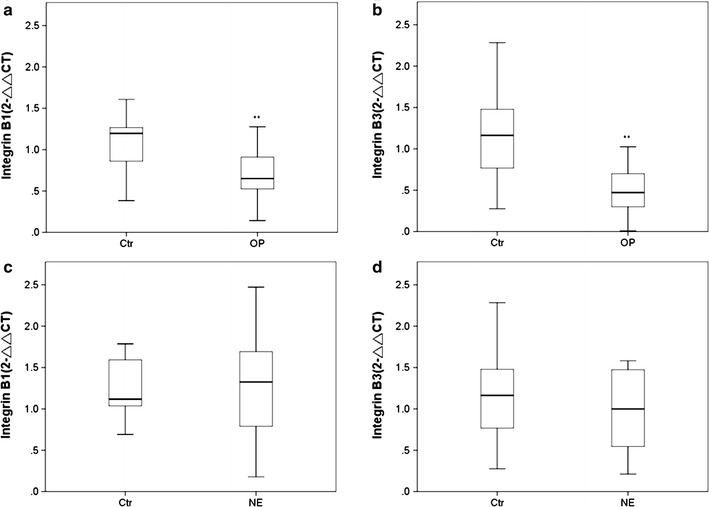
Expression levels of integrins β1 and β3 in endometrial cells were negatively associated with E_2_ levels in clinical samples. **a**, **b** Quantitative analysis of integrin β1 and β3 mRNA expression levels in the over physiological E2 group (OP). **c**, **d** Quantitative analysis of integrin β1 and β3 mRNA expression levels in the physiological effect group (NE). *Double asterisk* indicates significantly different from the control group, *P* < 0.01; *NS* not significantly different

It is known that integrin is one major member of extracellular matrix protein to modulate cell migration, cell–cell junction and embryo implantation. Integrin α1, α4, α5, β1 and β3 were reported to express in uterine endometria epithelia cells. We then measured the expression level of those integrins. There was no significantly difference expression level of integrin α1, α4 and α5 in all groups (data not shown). As illustrated by the qPCR results, there were significant differences between the relative levels of integrin β1 and β3 expression in the control group and high E_2_ group (*P* < 0.01; Fig. 1a, b; Additional file 1: Tables S2, S3); however, there was no significantly difference between the expression levels of these two indicators in the normal E_2_ and control groups (Fig. 1c, d). These results might be due to the blocking effect of E_2_ overexpression on integrin β1 and β3 transcription.

### Expression and surface distribution of integrin β3 on Ishikawa cells was regulated by E_2_

**Fig. 2:**
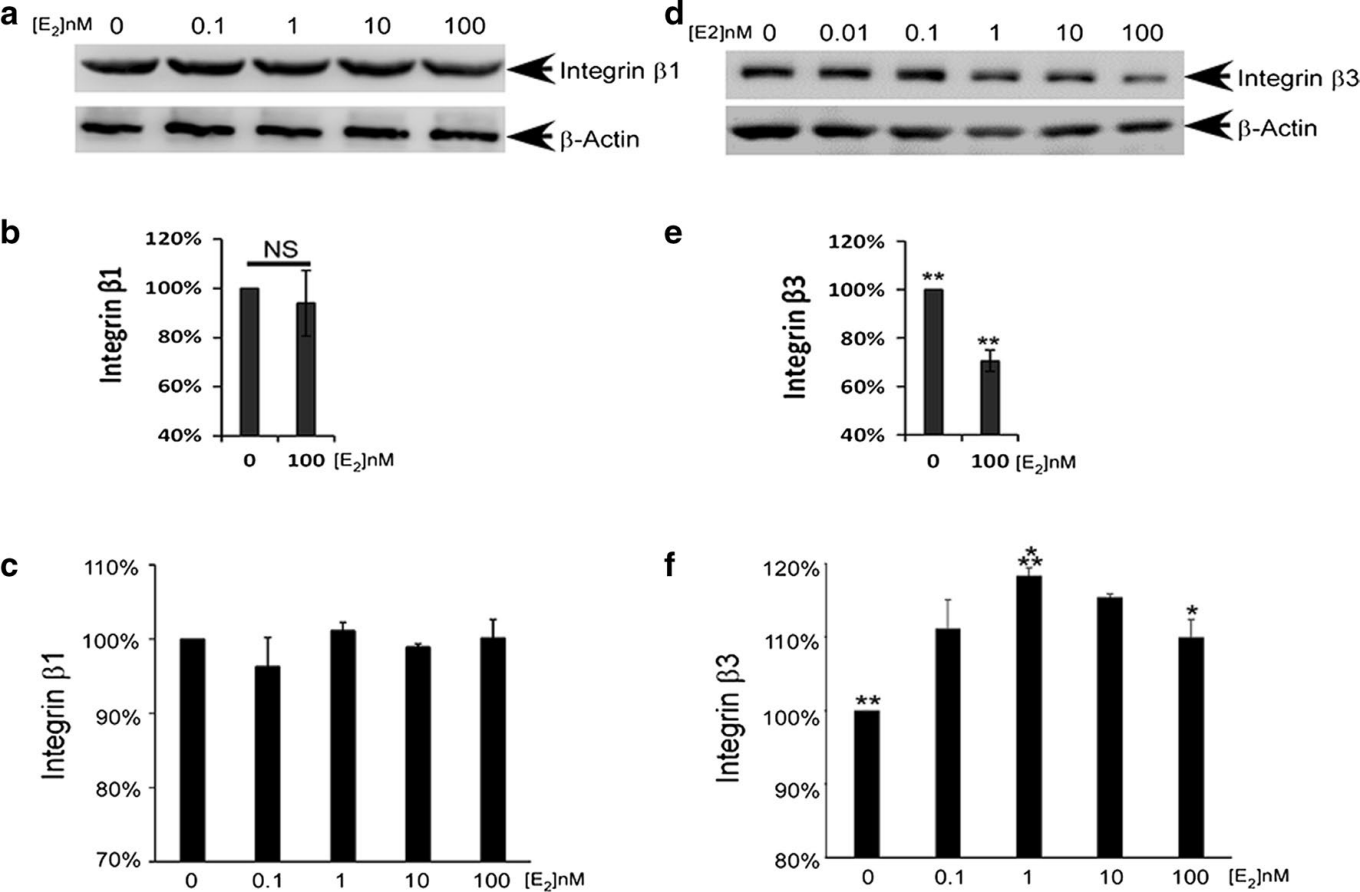
E_2_ enhanced the expression and distribution of integrin β3 on the plasma membranes of Ishikawa cells. **a** Representative image of integrin β1 expression as detected by western blotting. **b** Quantitative analysis integrin β1 expression as detected by western blotting. **c** Quantitative analysis of integrin β1 expression on the plasma membranes of Ishikawa cells as detected by flow cytometry. **d** Representative image of integrin β3 expression as detected by western blotting. **e** Quantitative analysis integrin β3 expression as detected by western blotting. **f** Quantitative analysis of integrin β3 distribution on the plasma membranes of Ishikawa cells as detected by flow cytometry. *Double asterisk* indicates significantly different from the control group, *P* < 0.01

To verify the regulatory effect of E_2_ on the expression of integrin β1 and β3, Ishikawa cells were exposed to different concentrations of E_2_; after which, the expression and distribution of the two integrins on the cell surface were detected. The results showed that contrary to our findings with clinical samples, different concentrations of E_2_ had little effect on the expression or distribution of integrin β1 on Ishikawa cells. We hypothesize that the E_2_ concentrations administered to Ishikawa cells needed to be much higher to induce any significant changes in integrin β1 expression and distribution (Fig. 2a–c; Additional file 1: Figure S1). In contrast, E_2_ administration produced remarkable alterations in the expression level and distribution of integrin β3 (Fig. 2d–f; Additional file 1: Figure S2). When the concentration of E_2_ was within a normal physiological range (<10 nM), the expression and distribution of integrin β3 was positively correlated with the concentration of E_2_; with 10 nM E_2_ exhibiting the most powerful promoting effect. However, when a higher than physiological concentration of E_2_ was administered (100 nM), there were dramatic declines in integrin β3 expression and distribution. Moreover, these declines were consistent with results obtained when using clinical samples.

### The expression and surface distribution of integrin β1 on Ishikawa cells was regulated by P4

**Fig. 3:**
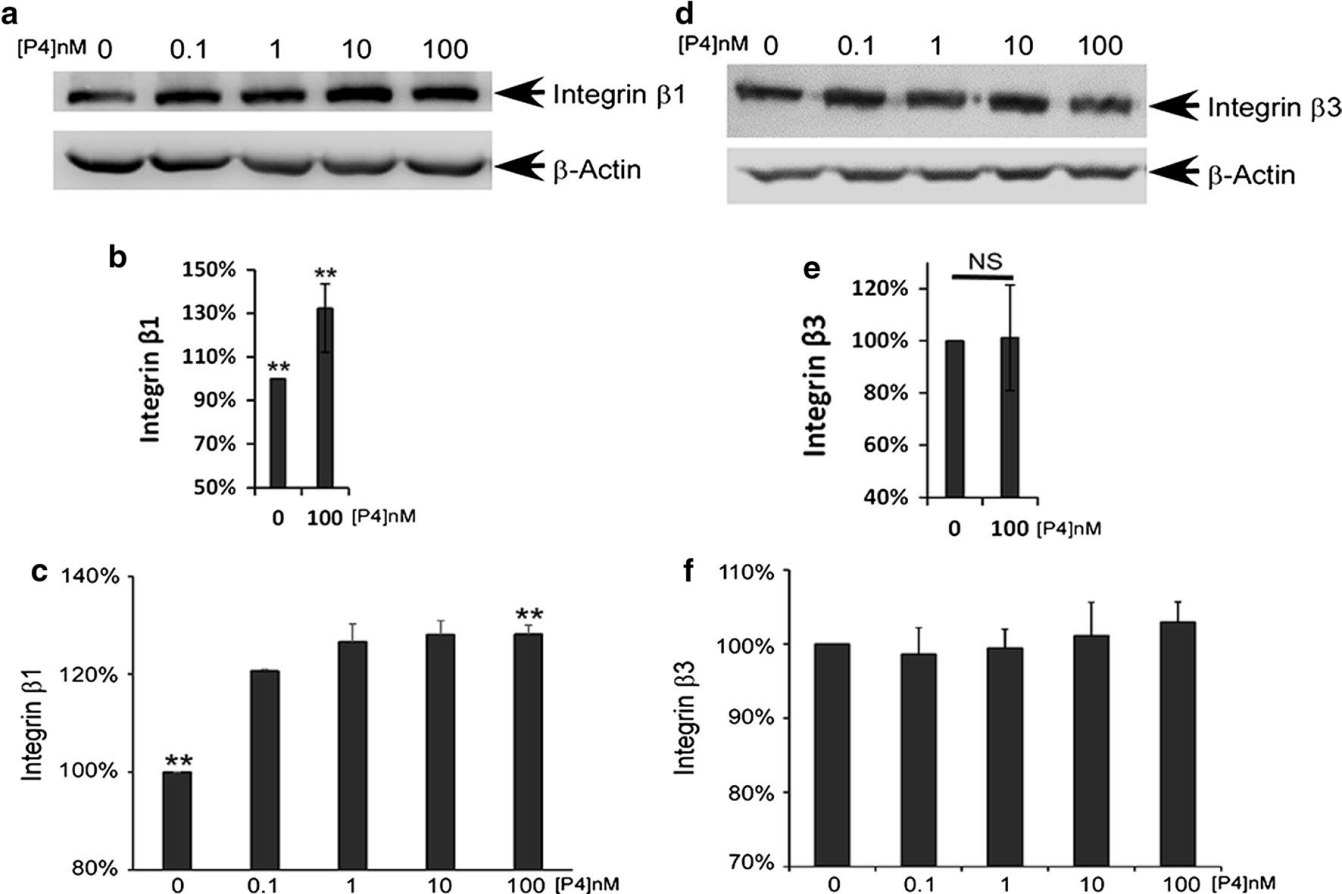
P4 enhanced the expression of integrin β1 and its distribution on the plasma membranes of Ishikawa cells. **a** Representative image of integrin β1 expression as detected by western blotting. **b** Quantitative analysis of integrin β1 expression as detected by western blotting. **c** Quantitative analysis of integrin β1 distribution on the plasma membranes of Ishikawa cells as detected by flow cytometry. **d** Representative image of integrin β3 expression as detected by western blotting. **e** Quantitative analysis of integrin β3 expression as detected by western blotting. **f** Quantitative analysis of integrin β3 distribution on the plasma membranes of Ishikawa cells as detected by flow cytometry. *Double asterisk* indicates significantly different from the control group, *P* < 0.01

We assessed the effect of P4 on the expression and surface distribution of integrins β1 and β3 in Ishikawa cells. Contrary to the effect of E_2_, administration of P4 had no influence on the activity of integrin β3; however, it had a positive effect on the production and distribution of integrin β1, even at a concentration of 100 nM (Fig. 3; Additional file 1: Figure S3, S4).

### Administration of P4 alleviated the negative effect of 100 nM E_2_ on integrin β3

**Fig. 4:**
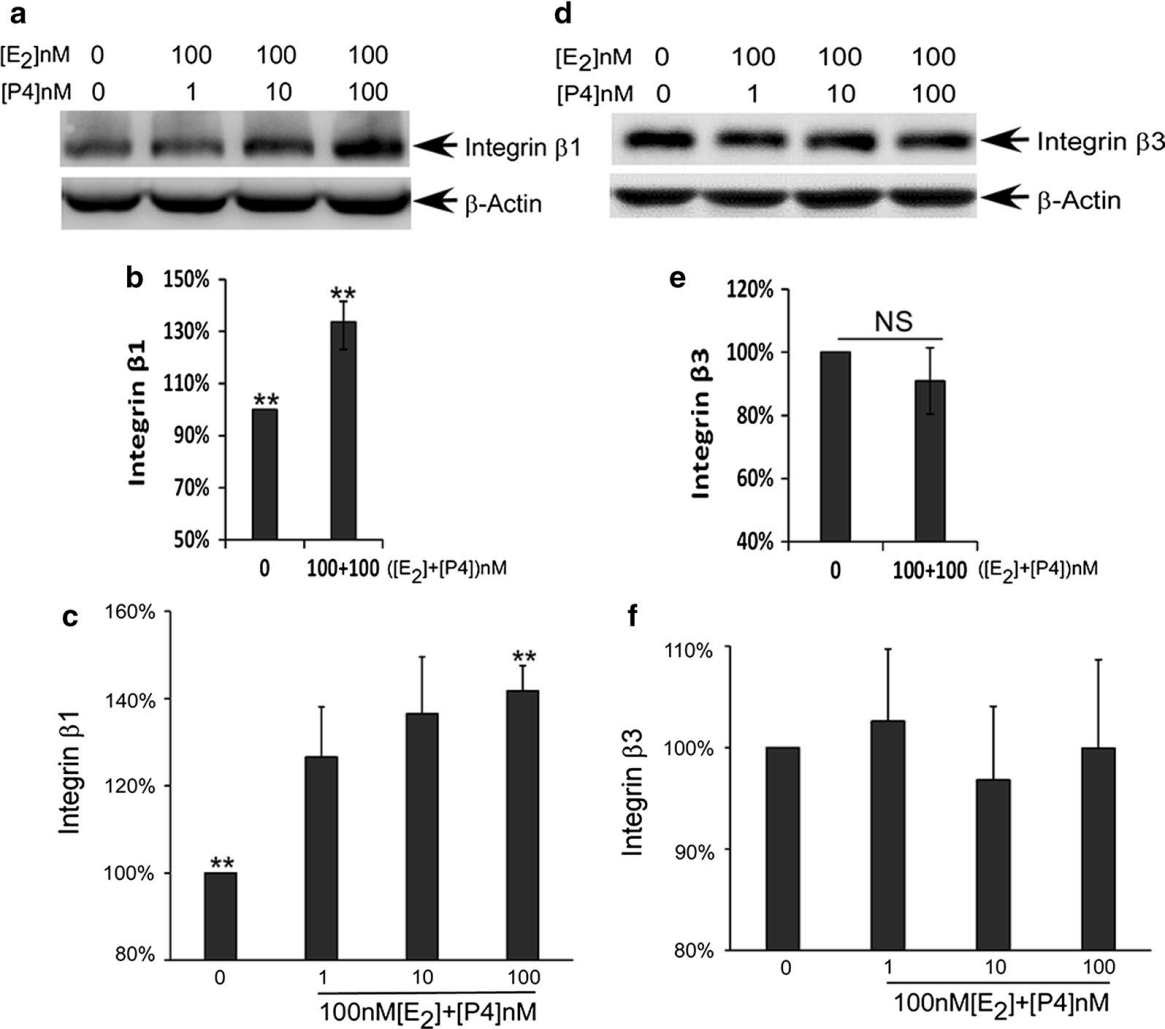
P4 eliminated E2-induced reductions in the expression and plasma membrane distribution of integrin β3 in Ishikawa cells. **a** Representative image of integrin β1 expression as detected by western blotting. **b** Quantitative analysis of integrin β1 expression as detected by western blotting. **c** Quantitative analysis of integrin β1 expression on the plasma membranes of Ishikawa cells as detected by flow cytometry. **d** Representative image of integrin β3 expression as detected by western blotting. **e** Quantitative analysis of integrin β3 expression as detected by western blotting. **f** Quantitative analysis of integrin β3 distribution on the plasma membranes of Ishikawa cells as detected by flow cytometry. *Double asterisk* indicates significantly different in three separate assays, *P* < 0.01

As demonstrated above, when administered at a concentration of 100 nM, E_2_ decreased the activity of integrin β3 in Ishikawa cells (Fig. 2d–f). However, when the cells were treated with 100 nM E_2_ plus different concentration of P4, the inhibitory effect of E_2_ on integrin β3 was eliminated (Fig. 4d–f; Additional file 1: Figure S5). The effects of concatenated administration on integrin β1 were similar to those obtained by administration of P4 alone (Fig. 4a–c; Additional file 1: Figure S6).

### P4 treatment increased the E_2_-induced reduction in implantation rate

**Fig. 5:**
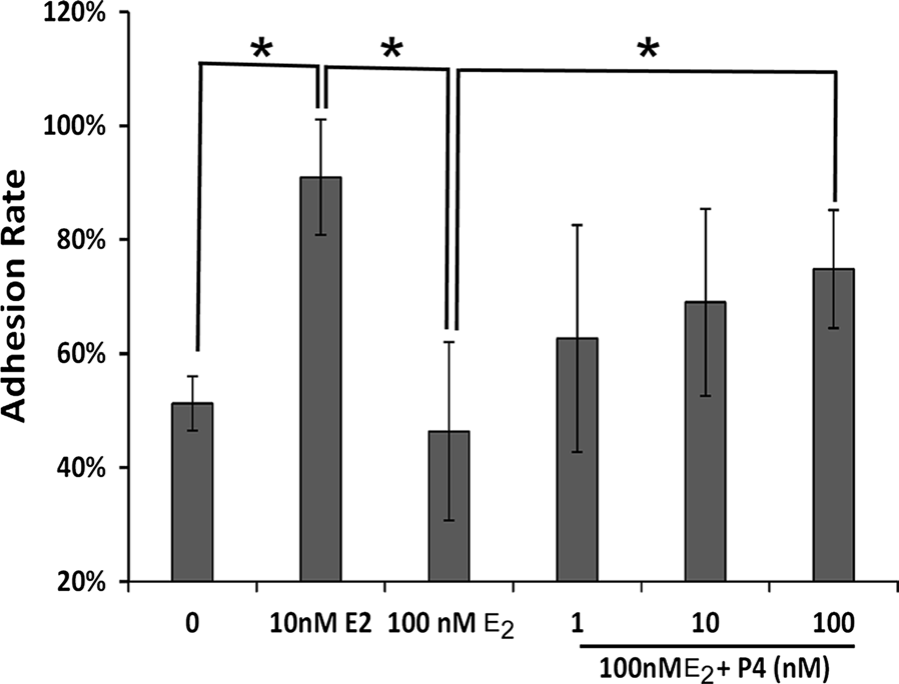
Both E_2_ and P4 could individually modulate the attachment of embryos onto Ishikawa cells. P4 recovered the decreased efficiency of embryo attachment produced by 100 nM E_2_. *Asterisk* indicates a statistically significant difference, *P* < 0.05

A 10 nM E_2_ concentration increased integrin β3 distribution on plasma membrane and enhanced embryo attachment when compared with a 0 nM E_2_ concentration (Fig. 5a; P < 0.01). However, a 100 nM E2 concentration reduced integrin β3 distribution on plasma membrane and inhibited embryo implantation compared with a10 nM E_2_ concentration (Fig. 5; P < 0.01). Treatment with P4 clearly ameliorated the negative effect of E_2_ on implantation in a dose-dependent manner, because P4 significantly enhanced the integrin β1 expression and distribution on plasma membrane (Fig. 4a–c). The difference in implantation rates obtained with 100 nM E_2_ treated cells and 100 nM P4 treated cells was statistically significant (*P* < 0.01).

## Discussion

Ovulation induction results in an increased [E_2_] and a lower embryo implantation rate. Moreover, E_2_ and P4 production are closely interconnected during the menstrual cycle. The respective role and molecular mechanism of each ovarian hormone has not been fully elucidated. Fortunately, the establishment of prime endometrial receptivity can serve as an original model in which to study the consequences of a single hormonal alteration on endometrial tissue [20]. In the current study, the respective effects of E_2_ and P4 on the combined expression patterns of integrins β1 and β3, which are biomarkers of uterine receptivity [21], were studied to assess their roles in establishing uterine receptivity in vitro.

In our clinical samples, E_2_ concentration >10 nM was associated with significantly decreased levels of integrin β3 expression. Based on our clinical investigations, the physiological concentrations of E_2_ range from 0.1 to 0.3 nM, and increase to ~3 nM during premenstrual and menstrual cycles. As previous in vitro studies have reported that a 10 nM E_2_ concentration had the strongest effect for inducing ERK1/2 and Akt phosphorylation [18], we selected the10 nM E_2_ concentration as our threshold for classifying clinical samples. Our investigations with clinical samples indicated that E_2_ concentrations >10 nM was associated with significantly decreased levels of integrin β1 expression. Our grouping strategy based on this criterion provided evidence that alterations in the E_2_ to P4 level had a marked influence on integrin expression. This effect might be related to a previous finding that only E_2_ concentrations >10 nM produced toxic effects in cells [18]. This idea had been further verified in a previous study in which 10 nM was shown to be the threshold concentration of E_2_ at which its positive effect on ovulation induction changed to a negative effect [22]. Moreover, findings in our current study also partially support this hypothesis. For example, our western blot studies showed no significant difference in integrin β3 expression when the E_2_ concentration was <10 nM (data not shown), while a 100 nM E_2_ concentration dramatically decreased integrin β3 distribution on plasma membrane when compared with a control E_2_ concentration.

The expression level of integrin and its plasma membrane distribution are one of the positive biomarkers of uterine receptivity [21]. In the present study, we screened the expression of integrins which were reported positive expression in human uterus. The expression level of integrin β1 and integrin β3 were regulated by blood E_2_ and P4 levels. Integrin β1 is constitutively expressed during the menstrual cycle and may be involved in the re-establishment of epithelialization following menstruation. Integrin β3 is expressed on a weekly basis by epithelial cells in proliferative endometrium; decreased endometrial expression of integrin β3 accompanied by reduced uterine receptivity has been verified in several pathological conditions [23]. Thus, the two indicators clearly reflect the effects of E_2_ and P4 on the establishment of uterine receptivity. In addition to determining how the E_2_ to P4 ratio affects integrin markers in clinical samples, we also established in vitro cell models for assessing the respective effects of E_2_ and P4 on integrin β3 and β1 expression and embryo attachment efficiency. Our in vitro results showed that 100 nM E_2_ significantly suppressed the production and distribution of integrin β3, while 100 nM P4 significantly enhanced the expression and surface distribution of integrin β1 and rescued the efficiency of embryo attachment inhibited by 100 nM E_2_. These results are indicative of the specific effect of each of these two hormones on different integrin members, suggest that is reasonable to concatenately use integrins β1 and β3 expression level and blood E2 and P4 level as biological markers for predicting uterine receptivity.

In addition to investigating how E_2_ and P4 levels respond to the respective functions of these two hormones, we also investigated the effects of treatment with different combinations of E_2_ and P4 concentrations. Our results showed that P4 attenuated the negative regulatory effect of E_2_ on integrin β3. It is known that P4 can inhibit the proliferative effect of E_2_ on endometrium by down-regulating the expression of E_2_ receptors [24]. While this antagonistic effect of P4 on E_2_ is generally believed to influence the completeness of the secretory transformation of endometrium [20]; during the embryo implantation process, a high E_2_ level will cause the window of uterine receptivity to rapidly close [1]. When considering the determinant role of E_2_ in specifying the duration of uterine receptivity for implantation, the use of P4 to regulate E_2_ levels might be justified as a method for improving IVF and ET results.

## Conclusions

In summary, our major finding was that increased E_2_ levels inhibited embryo implantation because they decreased the expression pattern of integrin β3. P4 markedly altered the expression pattern of integrin β1, and allowed it to rescue integrin β3 functions in the uterus. Because E_2_ and P4 appear to regulate the expression patterns of different integrins in specific manners, and when considering the respective functions of the two hormones, we recommend that any assessment of uterine receptivity should take the concatenate status of multiple biomarkers into consideration. We also hypothesize that antagonizing E_2_ by administration of P4 might be utilized as a method for improving the outcomes of techniques such as IVF and ET. Additional comprehensive studies regarding the E_2_-P4 balance and its effects on integrin family members will be performed to test our hypothesis.

## Additional file

**Additional file 1.** Additional figures and tables.

## Abbreviations

IVF: in vitro fertilization
ET: embryo transfer
P4: progesterone
ECM: extracellular matrix
E_2_: estradiol
IUD: intrauterine device
PCO: polycystic ovaries
LH: luteinizing hormone
GnRHa: gonadotropin-releasing hormone
FSH: follicle stimulating hormone
HCG: human chorionic gonadotropin
BMI: body mass index
PMSG: pregnant mare serum gonadotrophin
