# Supplementary material for: Integrins β1 and β3 are biomarkers of uterine condition for embryo transfer
Source: J Transl Med. 2016 Oct 26;14:303. doi: 10.1186/s12967-016-1052-0 (PMC5080684; doi:10.1186/s12967-016-1052-0)
Supplement: Supplementary file 1 — Additional file 1. Additional figures and tables. [file 12967_2016_1052_MOESM1_ESM.doc]

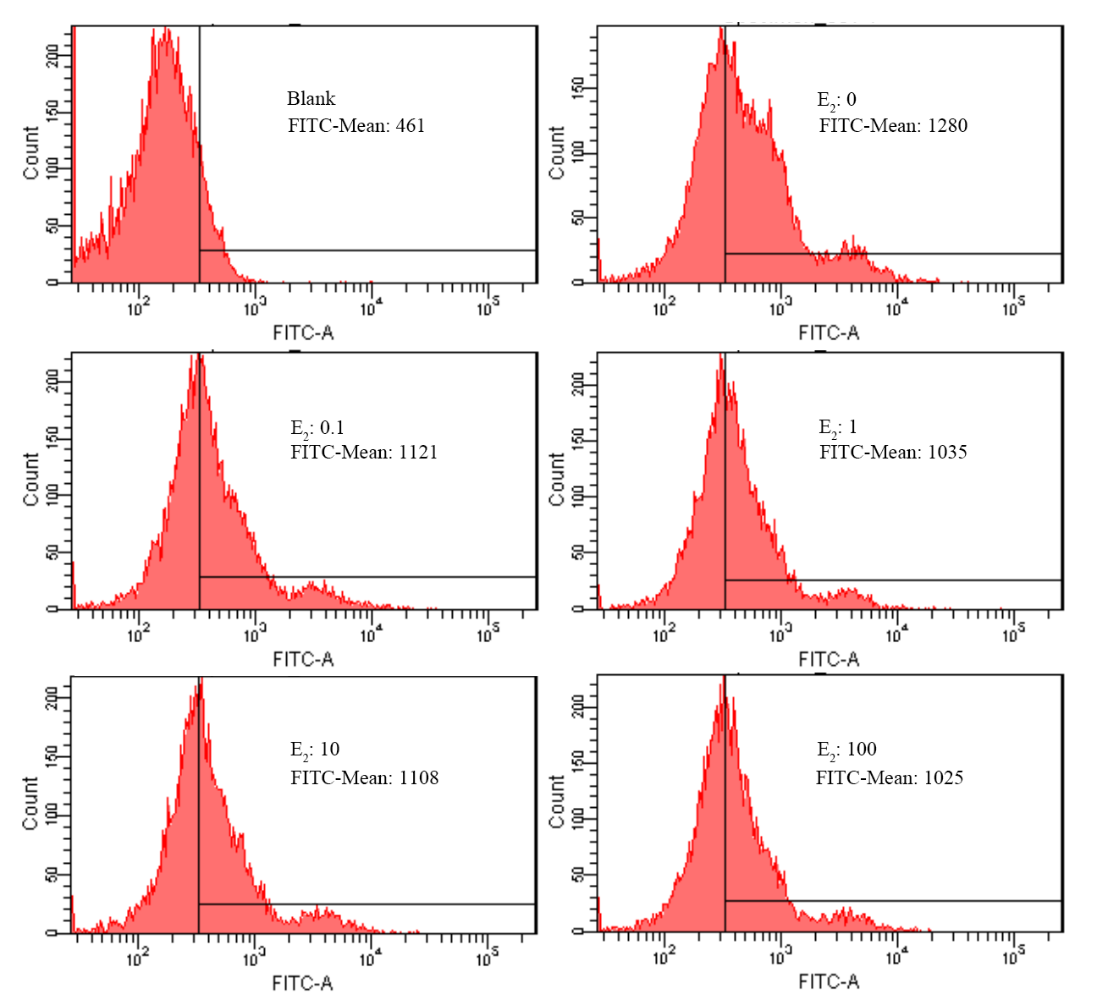


Sfig. 1. The quantity of Integrin β1 distributed on the plasma membranes of Ishikawa cells in different concentration of E_2_ as detected by flow cytometry.


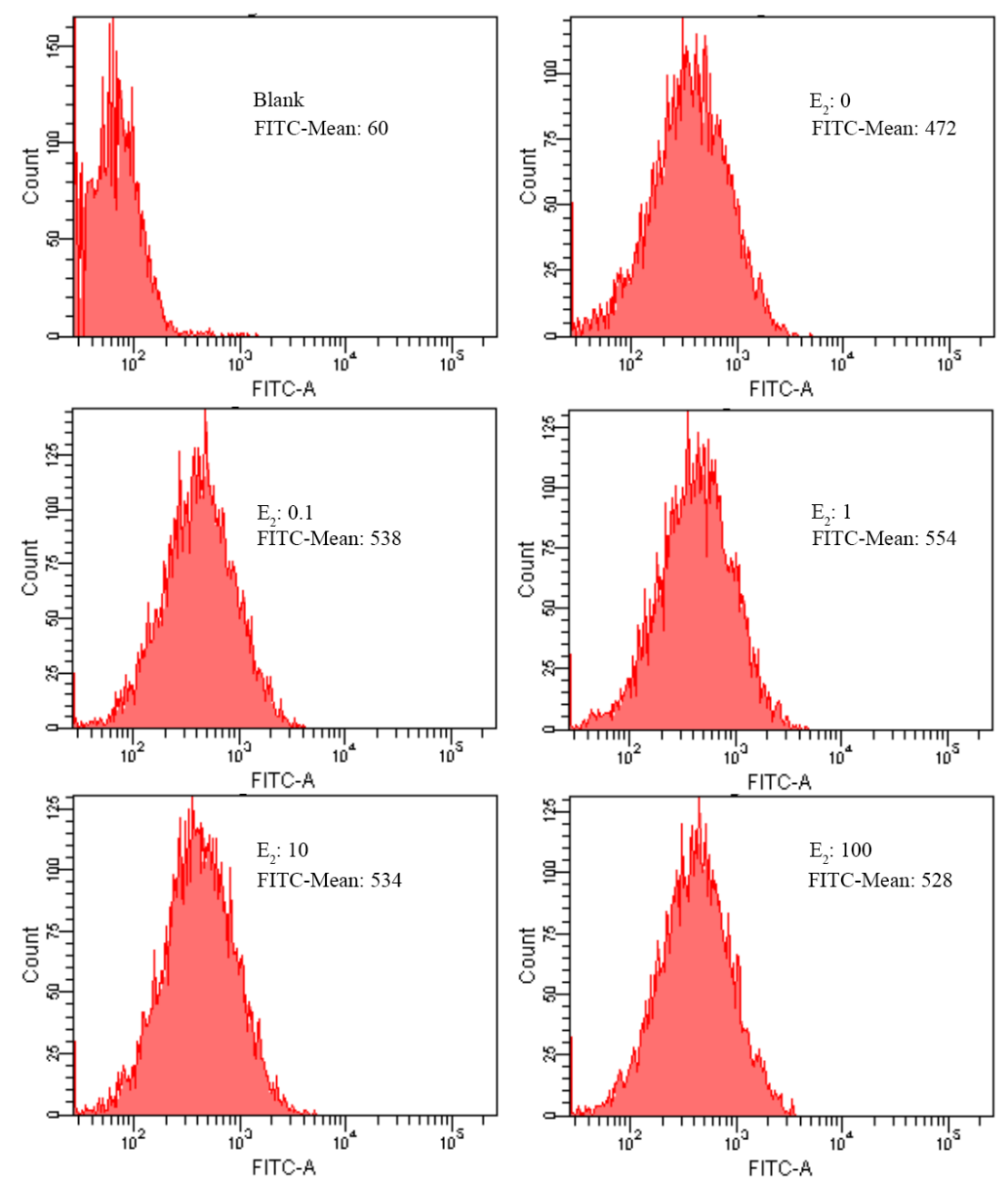


Sfig. 2. The quantity of Integrin β3 distributed on the plasma membranes of Ishikawa cells in different concentration of E_2_ as detected by flow cytometry.


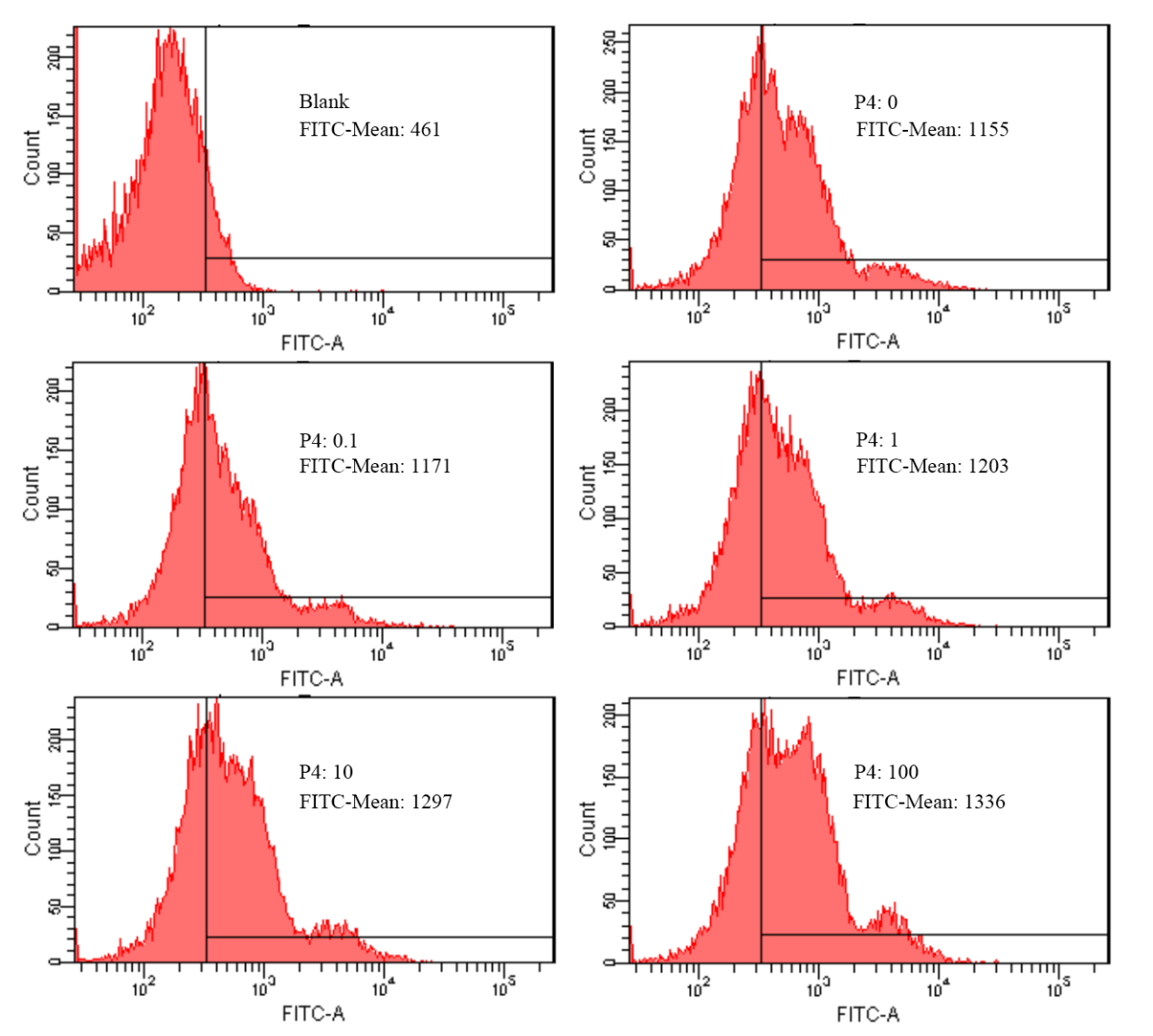


Sfig. 3. The quantity of Integrin β1 distributed on the plasma membranes of Ishikawa cells in different concentration of P4 as detected by flow cytometry.
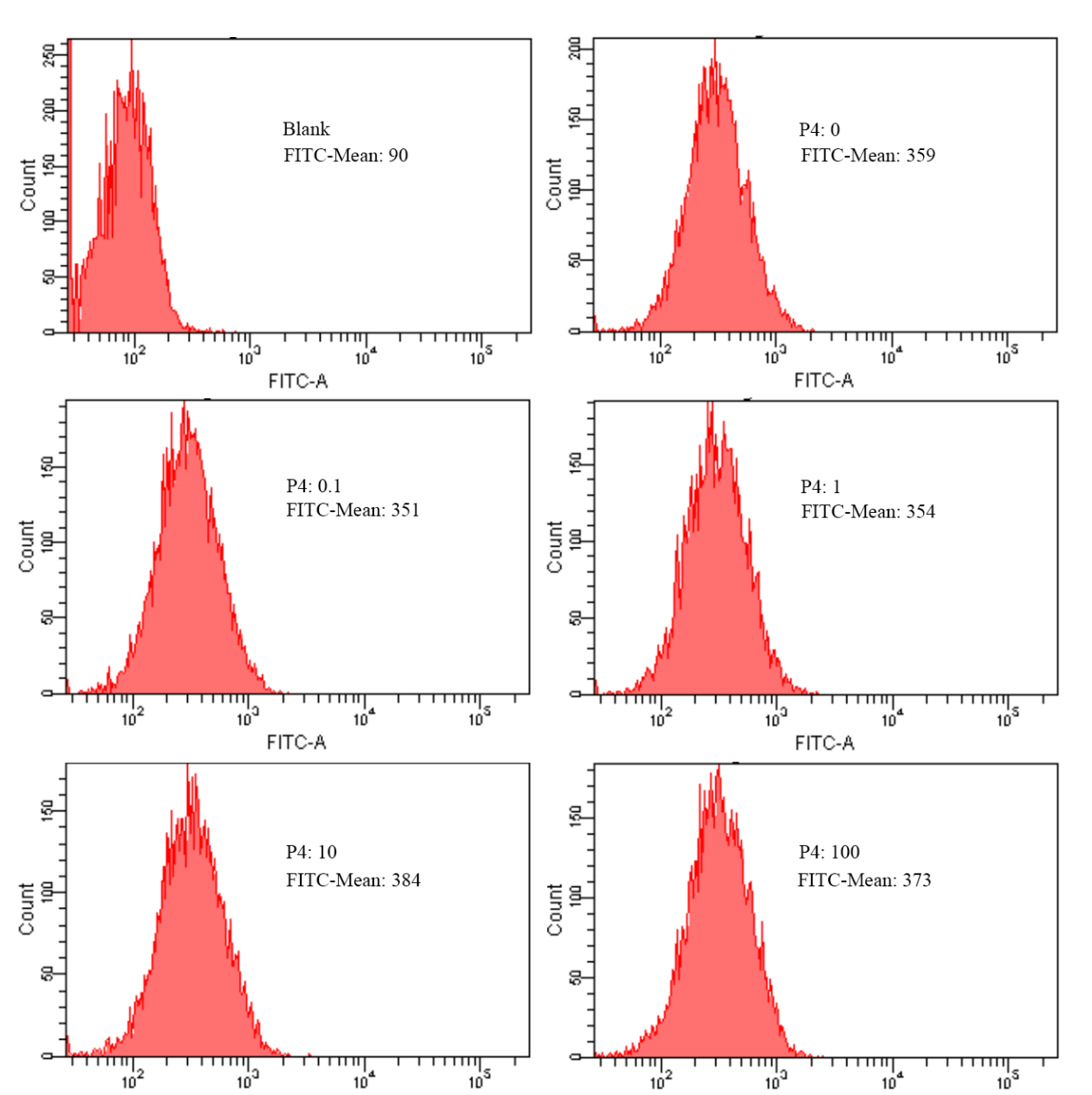


Sfig. 4. The quantity of Integrin β3 distributed on the plasma membranes of Ishikawa cells in different concentration of P4 as detected by flow cytometry.
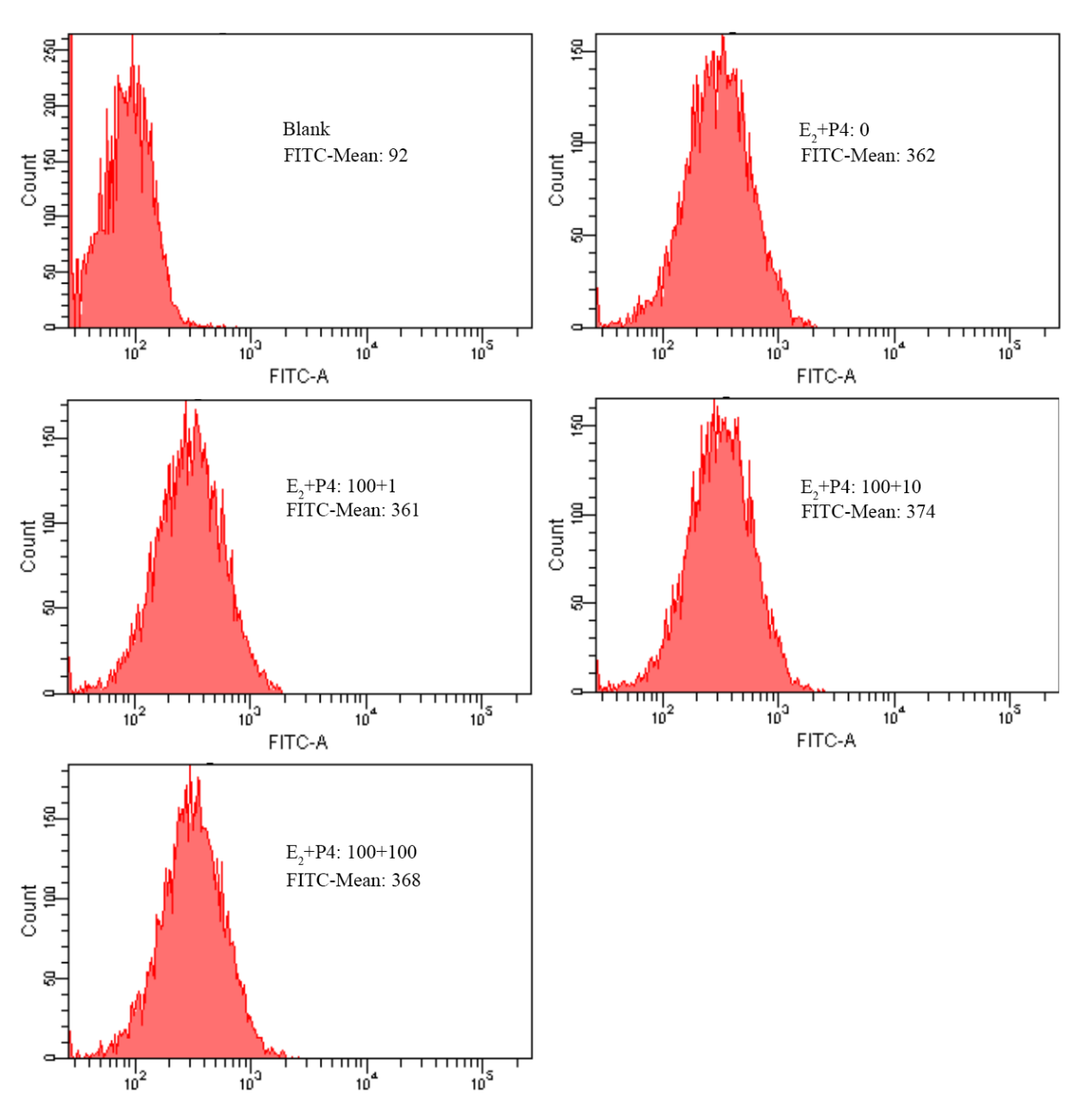


Sfig. 5. The quantity of Integrin β3 distributed on the plasma membranes of Ishikawa cells in high E_2_ with different concentration of P4 as detected by flow cytometry.
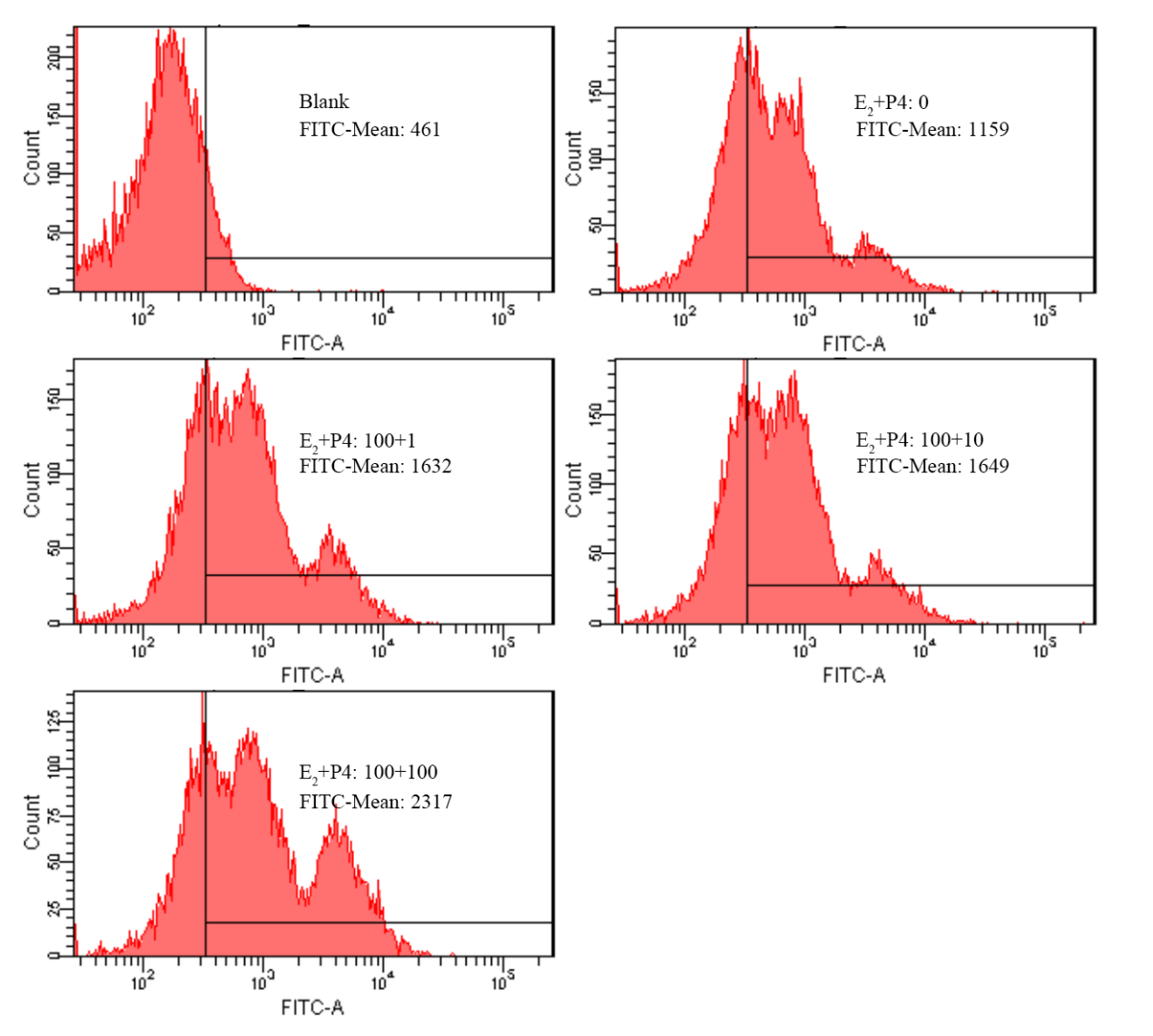
 Sfig. 6. The quantity of Integrin β1 distributed on the plasma membranes of Ishikawa cells in high E_2_ with different concentration of P4 as detected by flow cytometry.

Stab. 1. The characteristics of patients.

|  | Control Group  Ctr; n=13 | Normal Effect  NE; n=13 | Over Physiological  OP; n=18 |
| --- | --- | --- | --- |
| Infertile years | 1.92 ±0.21 | 2.30±0.24 | 1.78±0.19 |
| **Male Diagnosis** | Azospermia | Azospermia | Azospermia |
| **Female Diagnosis** |  |  |  |
| Polycystic ovaries | None | None | None |
| Endometriosis | None | None | None |
| Endometrial polyp | None | None | None |

Stab. 2 qPCR result for integrinβ1

| ID | GROUP | integrin B1 | | | B2M | | | △CT | △△CT | 2^-△△CT^ |
| --- | --- | --- | --- | --- | --- | --- | --- | --- | --- | --- |
|  |  | Test 1 | Test 2 | Average | Test 1 | Test 2 | Average |  |  |  |
| 1 | OP | 25.1700 | 25.0048 | 25.0874 | 23.1982 | 23.4293 | 23.3137 | 1.7736 | 0.3976 | 0.7591 |
| 2 | OP | 24.4439 | 24.6904 | 24.5671 | 22.4204 | 22.6690 | 22.5447 | 2.0224 | 0.6463 | 0.6389 |
| 3 | OP | 23.6683 | 23.7292 | 23.6987 | 22.6225 | 22.7253 | 22.6739 | 1.0248 | -0.3513 | 1.2757 |
| 4 | OP | 23.9635 | 24.1798 | 24.0716 | 21.7510 | 21.7325 | 21.7417 | 2.3299 | 0.9538 | 0.5163 |
| 5 | OP | 23.7599 | 23.7273 | 23.7436 | 21.8669 | 22.0001 | 21.9335 | 1.8101 | 0.4340 | 0.7402 |
| 6 | OP | 25.8548 | 26.1829 | 26.0188 | 23.9256 | 23.9111 | 23.9184 | 2.1005 | 0.7244 | 0.6052 |
| 7 | OP | 23.1524 | 23.1656 | 23.1590 | 21.8116 | 21.7591 | 21.7854 | 1.3736 | -0.0025 | 1.0017 |
| 8 | OP | 22.7963 | 22.9534 | 22.8748 | 20.6261 | 20.6790 | 20.6525 | 2.2223 | 0.8462 | 0.5562 |
| 9 | OP | 27.9633 | 27.8219 | 27.8926 | 23.6736 | 23.7189 | 23.6962 | 4.1964 | 2.8203 | 0.1416 |
| 10 | OP | 22.3397 | 22.5008 | 22.4203 | 19.4964 | 19.7471 | 19.6217 | 2.7985 | 1.4225 | 0.3731 |
| 11 | OP | 22.7937 | 22.5804 | 22.6871 | 19.9629 | 20.2054 | 20.0842 | 2.6029 | 1.2268 | 0.4273 |
| 12 | OP | 26.4628 | 26.2959 | 26.3794 | 24.1675 | 24.5506 | 24.3591 | 2.0203 | 0.6442 | 0.6398 |
| 13 | OP | 23.1532 | 22.9431 | 23.0481 | 21.4708 | 21.5999 | 21.5354 | 1.5128 | 0.1367 | 0.9096 |
| 14 | OP | 25.2848 | 25.1447 | 25.2147 | 22.8926 | 22.9236 | 22.9081 | 2.3066 | 0.9306 | 0.5247 |
| 15 | OP | 23.9647 | 23.9837 | 23.9742 | 22.4334 | 22.6610 | 22.5472 | 1.4270 | 0.0509 | 0.9653 |
| 16 | OP | 22.7162 | 22.7582 | 22.7372 | 20.8232 | 20.9483 | 20.8858 | 1.8514 | 0.4753 | 0.7193 |
| 17 | OP | 23.6771 | 23.6117 | 23.6444 | 22.2852 | 22.2619 | 22.2735 | 1.3709 | -0.0052 | 1.0036 |
| 18 | OP | 21.4512 | 21.4729 | 21.4621 | 19.3995 | 19.5802 | 19.4899 | 1.9722 | 0.5961 | 0.6615 |
| 19 | NE | 23.5178 | 23.2790 | 23.3984 | 21.8351 | 22.3124 | 22.0738 | 1.3246 | -0.0515 | 1.0363 |
| 20 | NE | 23.2106 | 22.9237 | 23.0671 | 21.3886 | 21.4574 | 21.4230 | 1.6441 | 0.2680 | 0.8305 |
| 21 | NE | 23.9900 | 24.5058 | 24.2479 | 22.8905 | 23.0010 | 22.9458 | 1.3021 | -0.0740 | 1.0526 |
| 22 | NE | 22.4606 | 22.4059 | 22.4332 | 22.1907 | 22.3197 | 22.2552 | 0.1780 | -1.1981 | 2.2943 |
| 23 | NE | 22.3996 | 22.4706 | 22.4351 | 21.5516 | 21.7391 | 21.6453 | 0.7898 | -0.5863 | 1.5014 |
| 24 | NE | 22.8037 | 22.9235 | 22.8636 | 21.7945 | 22.5772 | 22.1858 | 0.6778 | -0.6983 | 1.6226 |
| 25 | NE | 21.5240 | 21.4512 | 21.4876 | 20.3266 | 20.2965 | 20.3116 | 1.1760 | -0.2000 | 1.1487 |
| 26 | NE | 21.8455 | 21.6494 | 21.7475 | 20.8601 | 21.1816 | 21.0209 | 0.7266 | -0.6495 | 1.5686 |
| 27 | NE | 22.5157 | 22.8451 | 22.6804 | 21.2303 | 21.3396 | 21.2849 | 1.3955 | 0.0194 | 0.9867 |
| 28 | NE | 21.7378 | 21.7986 | 21.7682 | 19.2430 | 19.3500 | 19.2965 | 2.4716 | 1.0955 | 0.4680 |
| 29 | NE | 22.9733 | 23.1325 | 23.0529 | 21.3063 | 21.4144 | 21.3604 | 1.6925 | 0.3164 | 0.8030 |
| 30 | NE | 25.4292 | 25.5767 | 25.5029 | 23.2951 | 23.6079 | 23.4515 | 2.0514 | 0.6753 | 0.6262 |
| 31 | NE | 24.3657 | 24.4366 | 24.4011 | 22.5136 | 22.8403 | 22.6769 | 1.7242 | 0.3481 | 0.7856 |
| 32 | Ctr | 20.1481 | 20.4781 | 20.3131 | 19.2797 | 19.5955 | 19.4376 | 0.8755 | -0.5006 | 1.4148 |
| 33 | Ctr | 21.9039 | 22.0106 | 21.9573 | 21.2263 | 21.3052 | 21.2658 | 0.6915 | -0.6846 | 1.6072 |
| 34 | Ctr | 21.3977 | 21.4247 | 21.4112 | 20.5311 | 20.7162 | 20.6236 | 0.7876 | -0.5885 | 1.5037 |
| 35 | Ctr | 21.4558 | 21.6090 | 21.5324 | 20.0266 | 20.2677 | 20.1472 | 1.3853 | 0.0092 | 0.9937 |
| 36 | Ctr | 21.5402 | 21.7130 | 21.6266 | 20.5216 | 20.6076 | 20.5646 | 1.0620 | -0.3141 | 1.2432 |
| 37 | Ctr | 22.1935 | 21.9919 | 22.0927 | 20.8384 | 20.9564 | 20.8974 | 1.1953 | -0.1808 | 1.1335 |
| 38 | Ctr | 21.0142 | 21.0246 | 21.0194 | 19.8127 | 19.9934 | 19.9030 | 1.1164 | -0.2597 | 1.1972 |
| 39 | Ctr | 21.8426 | 22.1821 | 22.0124 | 20.3557 | 20.4825 | 20.4191 | 1.5933 | 0.2172 | 0.8602 |
| 40 | Ctr | 23.7622 | 23.9455 | 23.8539 | 22.6544 | 22.8188 | 22.7366 | 1.1173 | -0.2588 | 1.1965 |
| 41 | Ctr | 22.8835 | 23.0005 | 22.9420 | 21.8766 | 21.9353 | 21.9059 | 1.0361 | -0.3400 | 1.2658 |
| 42 | Ctr | 22.4536 | 22.4946 | 22.4741 | 19.9594 | 20.0258 | 19.9926 | 2.4815 | 1.1055 | 0.4648 |
| 43 | Ctr | 23.0156 | 23.3100 | 23.1628 | 21.3102 | 21.4426 | 21.3764 | 1.7864 | 0.4103 | 0.7525 |
| 44 | Ctr | 21.4135 | 21.4327 | 21.4231 | 18.5970 | 18.7269 | 18.6619 | 2.7611 | 1.3850 | 0.3829 |

Stab. 3 qPCR result for integrinβ3

|  |  | integrin B3 | | | B2M | | |  |  |  |
| --- | --- | --- | --- | --- | --- | --- | --- | --- | --- | --- |
| ID | GROUP | Test 1 | Test 2 | Average | Test 1 | Test 2 | Average | △CT | △△CT | 2-△△CT |
| 1 | OP | 32.1897 | 31.1386 | 31.6641 | 23.1982 | 23.4293 | 23.3137 | 8.3504 | 0.8396 | 0.5588 |
| 2 | OP | 42.7619 | 32.2794 | 37.5206 | 22.4204 | 22.6690 | 22.5447 | 14.9759 | 7.4651 | 0.0057 |
| 3 | OP | 31.3035 | 31.2096 | 31.2566 | 22.6225 | 22.7253 | 22.6739 | 8.5826 | 1.0718 | 0.4757 |
| 4 | OP | 29.9584 | 29.4080 | 29.6832 | 21.7510 | 21.7325 | 21.7417 | 7.9414 | 0.4306 | 0.7419 |
| 5 | OP | 29.9559 | 30.1610 | 30.0584 | 21.8669 | 22.0001 | 21.9335 | 8.1249 | 0.6141 | 0.6534 |
| 6 | OP | 33.5532 | 31.7388 | 32.6460 | 23.9256 | 23.9111 | 23.9184 | 8.7276 | 1.2168 | 0.4302 |
| 7 | OP | 29.9769 | 31.2348 | 30.6059 | 21.8116 | 21.7591 | 21.7854 | 8.8205 | 1.3097 | 0.4034 |
| 8 | OP | 28.1661 | 29.1946 | 28.6803 | 20.6261 | 20.6790 | 20.6525 | 8.0278 | 0.5170 | 0.6988 |
| 9 | OP | 30.9346 | 31.6633 | 31.2989 | 23.6736 | 23.7189 | 23.6962 | 7.6027 | 0.0919 | 0.9383 |
| 10 | OP | 29.6579 | 30.8113 | 30.2346 | 19.4964 | 19.7471 | 19.6217 | 10.6129 | 3.1021 | 0.1165 |
| 11 | OP | 30.5105 | 31.7679 | 31.1392 | 19.9629 | 20.2054 | 20.0842 | 11.0550 | 3.5442 | 0.0857 |
| 12 | OP | 30.7904 | 31.6824 | 31.2364 | 24.1675 | 24.5506 | 24.3591 | 6.8773 | -0.6335 | 1.5513 |
| 13 | OP | 32.0109 | 32.3967 | 32.2038 | 21.4708 | 21.5999 | 21.5354 | 10.6684 | 3.1576 | 0.1121 |
| 14 | OP | 32.4666 | 30.5719 | 31.5192 | 22.8926 | 22.9236 | 22.9081 | 8.6111 | 1.1003 | 0.4664 |
| 15 | OP | 31.2065 | 30.7812 | 30.9939 | 22.4334 | 22.6610 | 22.5472 | 8.4467 | 0.9359 | 0.5227 |
| 16 | OP | 28.4629 | 28.2589 | 28.3609 | 20.8232 | 20.9483 | 20.8858 | 7.4752 | -0.0357 | 1.0250 |
| 17 | OP | 30.5557 | 32.4853 | 31.5205 | 22.2852 | 22.2619 | 22.2735 | 9.2469 | 1.7361 | 0.3002 |
| 18 | OP | 29.1500 | 28.2991 | 28.7246 | 19.3995 | 19.5802 | 19.4899 | 9.2347 | 1.7239 | 0.3027 |
| 19 | NE | 31.6409 | 30.4634 | 31.0521 | 21.8351 | 22.3124 | 22.0738 | 8.9784 | 1.4675 | 0.3616 |
| 20 | NE | 29.9453 | 30.2544 | 30.0999 | 21.3886 | 21.4574 | 21.4230 | 8.6768 | 1.1660 | 0.4456 |
| 21 | NE | 31.6653 | 28.7959 | 30.2306 | 22.8905 | 23.0010 | 22.9458 | 7.2848 | -0.2260 | 1.1696 |
| 22 | NE | 27.9282 | 28.4224 | 28.1753 | 22.1907 | 22.3197 | 22.2552 | 5.9201 | -1.5907 | 3.0120 |
| 23 | NE | 28.5301 | 28.4615 | 28.4958 | 21.5516 | 21.7391 | 21.6453 | 6.8505 | -0.6603 | 1.5805 |
| 24 | NE | 29.8705 | 29.5259 | 29.6982 | 21.7945 | 22.5772 | 22.1858 | 7.5124 | 0.0015 | 0.9989 |
| 25 | NE | 28.4606 | 28.3531 | 28.4069 | 20.3266 | 20.2965 | 20.3116 | 8.0953 | 0.5845 | 0.6669 |
| 26 | NE | 28.7789 | 29.3267 | 29.0528 | 20.8601 | 21.1816 | 21.0209 | 8.0319 | 0.5211 | 0.6968 |
| 27 | NE | 29.8690 | 29.4719 | 29.6704 | 21.2303 | 21.3396 | 21.2849 | 8.3855 | 0.8747 | 0.5454 |
| 28 | NE | 28.7130 | 29.3840 | 29.0485 | 19.2430 | 19.3500 | 19.2965 | 9.7520 | 2.2411 | 0.2115 |
| 29 | NE | 28.4069 | 28.3305 | 28.3687 | 21.3063 | 21.4144 | 21.3604 | 7.0083 | -0.5025 | 1.4166 |
| 30 | NE | 30.3034 | 30.5027 | 30.4030 | 23.2951 | 23.6079 | 23.4515 | 6.9515 | -0.5593 | 1.4736 |
| 31 | NE | 28.6154 | 28.6471 | 28.6312 | 22.5136 | 22.8403 | 22.6769 | 5.9543 | -1.5565 | 2.9415 |
| 32 | Ctr | 26.5028 | 26.6219 | 26.5623 | 19.2797 | 19.5955 | 19.4376 | 7.1247 | -0.3861 | 1.3068 |
| 33 | Ctr | 28.3856 | 28.0040 | 28.1948 | 21.2263 | 21.3052 | 21.2658 | 6.9290 | -0.5818 | 1.4967 |
| 34 | Ctr | 27.8755 | 27.9589 | 27.9172 | 20.5311 | 20.7162 | 20.6236 | 7.2936 | -0.2173 | 1.1625 |
| 35 | Ctr | 28.7794 | 28.8375 | 28.8085 | 20.0266 | 20.2677 | 20.1472 | 8.6613 | 1.1505 | 0.4505 |
| 36 | Ctr | 27.3557 | 27.6655 | 27.5106 | 20.5216 | 20.6076 | 20.5646 | 6.9460 | -0.5648 | 1.4792 |
| 37 | Ctr | 28.7657 | 28.8126 | 28.7891 | 20.8384 | 20.9564 | 20.8974 | 7.8917 | 0.3809 | 0.7679 |
| 38 | Ctr | 28.2612 | 28.3670 | 28.3141 | 19.8127 | 19.9934 | 19.9030 | 8.4111 | 0.9003 | 0.5358 |
| 39 | Ctr | 26.9078 | 28.3887 | 27.6483 | 20.3557 | 20.4825 | 20.4191 | 7.2292 | -0.2816 | 1.2156 |
| 40 | Ctr | 28.4374 | 29.6734 | 29.0554 | 22.6544 | 22.8188 | 22.7366 | 6.3189 | -1.1920 | 2.2846 |
| 41 | Ctr | 28.5033 | 28.4980 | 28.5006 | 21.8766 | 21.9353 | 21.9059 | 6.5947 | -0.9161 | 1.8870 |
| 42 | Ctr | 26.9746 | 28.1470 | 27.5608 | 19.9594 | 20.0258 | 19.9926 | 7.5682 | 0.0574 | 0.9610 |
| 43 | Ctr | 28.7178 | 28.6389 | 28.6784 | 21.3102 | 21.4426 | 21.3764 | 7.3020 | -0.2088 | 1.1558 |
| 44 | Ctr | 27.8431 | 28.2211 | 28.0321 | 18.5970 | 18.7269 | 18.6619 | 9.3702 | 1.8594 | 0.2756 |
